# Zebrafish brain atlases: a collective effort for a tiny vertebrate brain

**DOI:** 10.1117/1.NPh.10.4.044409

**Published:** 2023-09-30

**Authors:** Antoine Légaré, Mado Lemieux, Patrick Desrosiers, Paul De Koninck

**Affiliations:** a CERVO Brain Research Center, Québec, Québec, Canada; b Université Laval, Department of Physics, Engineering Physics and Optics, Québec, Québec, Canada; c Université Laval, Department of Biochemistry, Microbiology and Bio-informatics, Québec, Québec, Canada

**Keywords:** zebrafish, brain atlas, calcium imaging, whole-brain imaging, circuit reconstruction, open-access datasets

## Abstract

In the past two decades, digital brain atlases have emerged as essential tools for sharing and integrating complex neuroscience datasets. Concurrently, the larval zebrafish has become a prominent vertebrate model offering a strategic compromise for brain size, complexity, transparency, optogenetic access, and behavior. We provide a brief overview of digital atlases recently developed for the larval zebrafish brain, intersecting neuroanatomical information, gene expression patterns, and connectivity. These atlases are becoming pivotal by centralizing large datasets while supporting the generation of circuit hypotheses as functional measurements can be registered into an atlas’ standard coordinate system to interrogate its structural database. As challenges persist in mapping neural circuits and incorporating functional measurements into zebrafish atlases, we emphasize the importance of collaborative efforts and standardized protocols to expand these resources to crack the complex codes of neuronal activity guiding behavior in this tiny vertebrate brain.

## 1 Introduction

Tremendous progress in photonics, optogenetics, and computational methods over the last two decades has endowed the field of neuroscience with a wealth of large and complex datasets in which structural, physiological, and behavioral measurements are simultaneously acquired from animal models.^1^ From these diverse experimental vantage points, our understanding of neuronal computations spanning several brain regions has grown remarkably. However, as the widespread accessibility of genetically encoded fluorescent sensors and laser-scanning microscopy techniques has rendered large neurophysiological recordings an increasing commodity, we face the collective challenge of standardizing and comparing ever-elaborate experiments and results obtained across research groups. Numerous imaging protocols, data analysis pipelines, and file-sharing systems have been proposed in recent years and adopted in some cases by large institutions, providing much-needed guidelines for the homogeneity of neurophysiological data.^2^^,^^3^

At the center point of these standardization efforts, digital brain atlases have been developed in several animal models,^4^^–^^8^ becoming highly invaluable tools in neuroscience. By providing a standardized coordinate space into which experiments can be mapped, digital atlases have become hubs for sharing data across imaging modalities and research groups. The efforts conducted by the Allen Institute to map the mouse brain represent a notable example of large-scale endeavors to compile anatomical parcellations, cellular architecture, connectivity, and gene expression into a single reference space.^9^ Most neuroscientists can benefit from these large datasets; however, the incorporation of new measurements at whole-brain scale remains hardly feasible for individual labs as the exhaustive sampling of a single imaging modality requires complex tissue clearing protocols or thousands of automated slicing and imaging experiments that rely on sophisticated infrastructure. By contrast, the larval zebrafish offers a currently unique opportunity as a model system: that of imaging an entire vertebrate brain within minutes, *in vivo*, using conventional optical microscopy approaches. Striking a good balance among size, optical and genetic accessibility, and complexity has brought the zebrafish into the spotlight as a highly valuable model system for investigating brain-wide neuronal dynamics across various sensorimotor contexts.^10^^–^^12^ To complement these functional studies, several zebrafish brain atlases have been built. We give a brief overview of these resources while focusing on the efforts made at the larval stage, when whole-brain sampling remains widely achievable. We underline the importance of leveraging these continuously growing resources and discuss the possibility of collectively painting a detailed picture of a small vertebrate brain at the microscopic scale through standardized experiments and data sharing.

## 2 Atlases of the Larval Zebrafish Brain

Many pioneering studies have firmly established the larval zebrafish as a well-suited model for studying whole-brain neuronal activity using calcium imaging while preserving cellular resolution over roughly $10^{5}$ neurons.^10^^,^^13^ To build comprehensive descriptions of neuronal circuits, however, activity measurements across the brain ideally have to be overlaid on neuroanatomical measurements. A handful of digital atlases have been developed at the larval stage to provide a common anatomical and numerical ground for whole-brain calcium imaging studies (Table 1). These atlases provide considerable anatomical information, including brain region delimitations, spatial distributions of gene expression, and connectivity between regions.

**Table 1:** Brain atlases of the zebrafish, with their various attributes listed as of September 2023. Hyperlinks are embedded in the atlas names to access web databases.

Atlas	Stage	Attributes	Publications
ViBE-Z	3 dpf	17 labels (confocal) and 73 brain regions	Ronneberger et al.^14^
Z-Brain	6 dpf	29 labels (confocal), ∼294 brain regions, EM data, and circuit explorer	Randlett et al. ^16^
			Hildebrand et al.^22^
			Vohra et al.^23^
Zebrafish Brain Browser	6 dpf	Hundreds of labels (confocal), ∼294 brain regions (Z-Brain), and 3D visualization	Marquart et al.^17^
			Marquart et al.^18^
MapZebrain	6 dpf	>700 labels (confocal), ∼70 brain regions, neuronal morphology and connectivity, and EM data	Kunst et al.^19^
			Svara et al.^24^
			Shainer et al.^21^
MRI Atlas	Adult	High-resolution magnetic resonance histology and 53 brain regions	Ullmann et al.^25^
AZBA	Adult	10 labels (tissue clearing + lightsheet) and >200 brain regions	Kenney et al.^26^

The first digital atlas of the larval zebrafish brain, the virtual brain explorer (ViBE-Z), was built for 3 days post fertilization (DPF) larvae, a period in which larvae are fully transparent and many circuit elements are already established.^14^ Using custom landmark alignment, elastic registration, and intensity correction algorithms, the authors assembled 17 co-registered labels from multiple fish in a standardized volume using confocal microscopy. Drawing from similar work in *Drosophila*,^15^ this work established a standard framework for atlas building in the zebrafish, as multiple elements of this first iteration, such as the use of a second structural staining for alignment, manual segmentations of anatomical regions, and an open-access web portal, have been key elements of the subsequent resources.

To support studies focusing on the 5 to 7 DPF period, when larvae notably begin to hunt, the Z-Brain Atlas was built from hundreds of confocal brain stacks of fixed 6 DPF larvae.^16^ The authors compiled a total of 29 labels using a bridge channel of total ERK expression, as well as manual segmentations of 294 anatomical regions. The same year, the Zebrafish Brain Browser was published, hosting a catalog of 109 live-imaged transgenic lines in a 3D web interface.^17^ The authors used multi-channel registration between their reference volume and the Z-Brain atlas to merge both datasets, highlighting the symbiotic and complementary nature of these resources when data are acquired through similar protocols.^18^

More recently, the Max Planck Zebrafish Brain Atlas (mapZebrain) was built at 6 DPF using similar methods, combining over 100 structural markers and further demonstrating the compatibility of these resources by aligning all 6 DPF digital atlases up to that point.^19^ The authors also introduced novel connectivity measurements by registering over 2000 stochastically labeled single-neuron tracings into the reference volume. Although these light microscopy (LM) measurements of neuronal morphology provide no synaptic information, they give crucial insight into connectivity patterns at the mesoscopic scale while constraining computational models of neuronal dynamics across brain regions.^20^ The same group recently expanded their atlas by incorporating over 200 gene expression markers obtained with fluorescence *in situ* hybridization, opening the door to high-throughput mapping of gene expression using commercially available and replicable protocols.^21^ These resources have grown continuously since their instigation, with new data being incorporated by other research groups, highlighting the importance of collectively expanding the existing databases.

## 3 Workflow of Mapping the Larval Zebrafish Brain

**Fig. 1:**
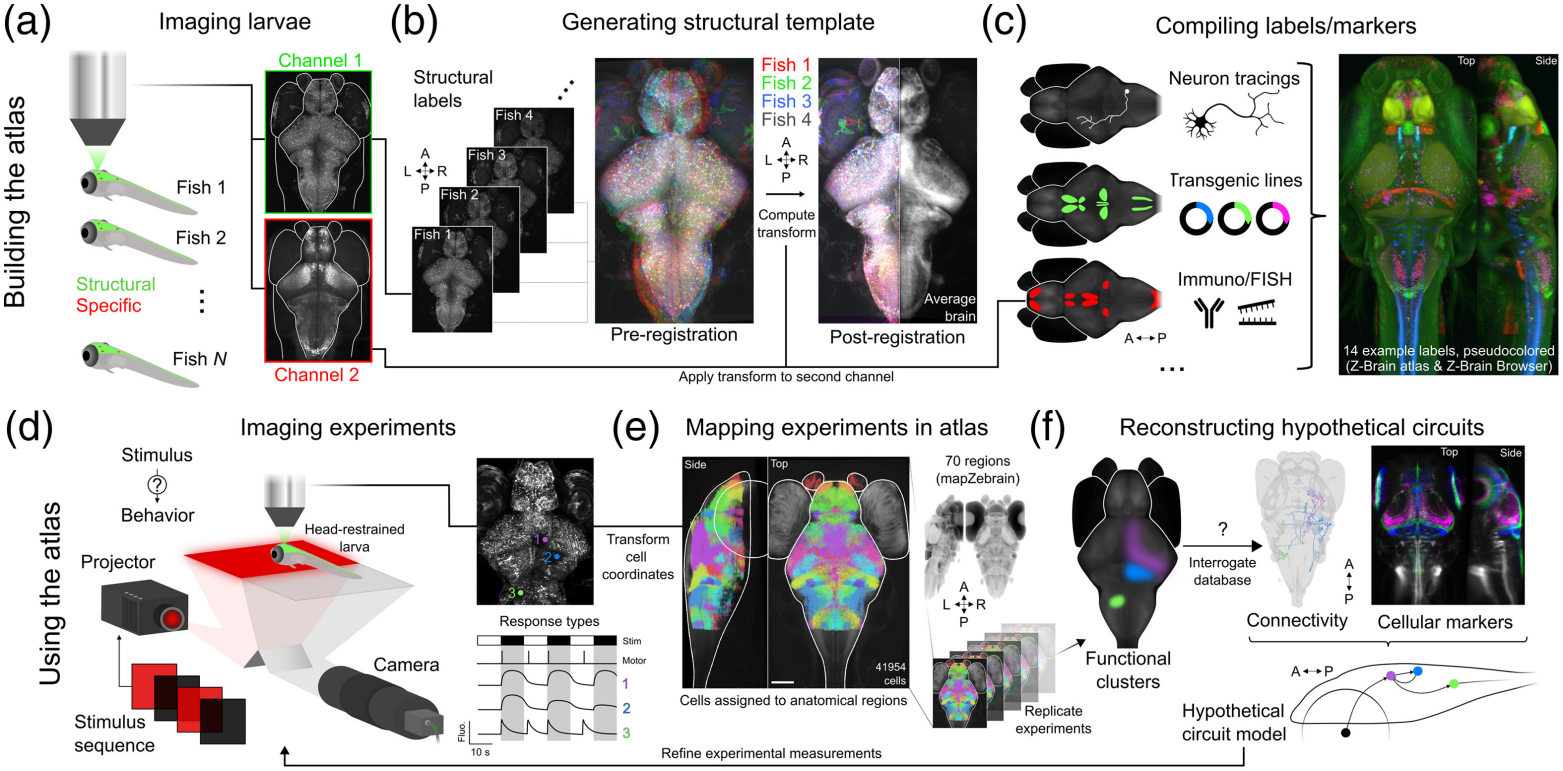
Workflow of brain mapping in the larval zebrafish. (a) Serial dual-color imaging of multiple fluorescently labeled larvae (channel 1, Tg (elavl3: H2b-GCaMP6s) and channel 2, anti-tyrosine hydroxylase immunolabeling). (b) Registration of multiple structural labels to generate a template brain (nuclear GCaMP channel). (c) Alignment of multiple structural labels onto a template brain to generate an atlas; background images and markers taken from Z-Brain Atlas. (d) Simultaneous visual stimulation, behavior, and whole-brain calcium imaging; neuronal response profiles are identified from the data. (e) Neuron centroids are mapped into distinct anatomical regions of the atlas; background image and regions taken from mapZebrain atlas. (f) Following functional measurements across fish, functional maps are compared with structural data to identify putative circuit models that can then be validated experimentally; neurons and markers taken from mapZebrain atlas. Panels (d)–(f) reflect purely hypothetical experiments and circuits. Orientations: A, anterior; P, posterior; L, left; and R, right.

The advantageous properties of the larval stage—most notably, optical accessibility, rapid development, and small size—have facilitated a high-throughput approach for imaging structural markers across the larval fish brain to build atlases, using a combination of immunolabelings, *in situ* hybridizations, or genetically encoded fluorescent indicators [Fig. 1(c)]. Crucially, the exhaustive sampling—that is, imaging the entire brain volume while maintaining a high spatial resolution—of these labels is considerably more accessible in larval zebrafish than in larger animal models as the whole brain volume can be stained using whole-mount protocols or imaged directly *in vivo* in transgenic lines expressing fluorescent markers/sensors. Multiple larvae can be embedded in low melting point agarose in a single imaging chamber and serially imaged using one or two confocal volumes per brain [Fig. 1(a)]. This workflow allows for the relatively rapid collection of large databases of brain scans in which specific neuronal labels are co-labeled with a brain-wide structural marker (such as nuclear, ERK, or synapsin stainings) that highlights important landmarks potentially missing from more specific, sparser expression patterns.

The alignment between different imaging volumes is typically achieved using nonrigid image registration algorithms from the commonly used Computational Morphometry Toolkit^27^ or Advanced Normalization Tools.^28^^,^^29^ Of note, the latter package was shown to minimize local deformations,^18^ thus being prioritized for more recent atlases. The cellular precision of the alignment procedure has been well demonstrated; it is capable of compensating for variability in brain size, morphology, and 3D orientation. It reliably preserves cellular morphology and, in some cases, stereotyped cell positions across individuals,^16^^,^^18^ though differences in neuronal migration might spatially offset smaller cell clusters within larger anatomical structures. Before specific labels can be aligned onto each other, however, an initial template brain of the main structural marker has to be established as a reference for subsequent alignments [Fig. 1(b)]. The standard and most robust approach is to compute an iterative average of multiple brains using a multivariate template alignment, which eliminates any bias introduced when hand-picking a single reference brain from a representative specimen.^30^ After the initial template is generated, all structural markers are transformed into the reference volume and then averaged to obtain smooth spatial distributions of transgene expression. This process can then be repeated independently and in parallel until hundreds or thousands of markers are compiled into a single standardized volume [Fig. 1(c)].

From the perspective of an atlas user, the library of structural markers offers multiple entry points for aligning experimental data into the standard coordinate space of an atlas [Fig. 1(d)]. After computing transforms from raw data to the atlas using registration algorithms, landmarks or cell coordinates from calcium imaging experiments can be transformed into the atlas referential [Fig. 1(e)]. They can then be used to index anatomical regions precisely and interrogate the database to identify putative genetically defined cell populations [Fig. 1(f)]. Note that all of the aforementioned steps for building or utilizing a digital atlas involve widely available and well-documented tools, as well as datasets of tractable size. As such, the brain mapping pipeline is readily achievable in the zebrafish community, holding promises for the next decade of collaborative work in expanding these essential resources.

## 4 Perspectives

Atlases have become essential tools that support the interpretation and comparison of neurophysiological data, enabling the generation of circuit hypotheses by overlapping functional measurements, such as calcium or voltage^31^ imaging, on a catalog of spatially defined structural measurements, such as gene expression or connectivity [Fig. 1(f)]. A transformative development in the effort of fusing functional and anatomical data in larval zebrafish has been the creation of broadly used transgenic lines expressing GCaMP in every neuron,^13^^,^^32^ tremendously facilitating the brain-wide mapping of neuronal responses and subsequent alignment of calcium imaging results on the atlases. However, new challenges are encountered when increasingly numerous and complementary structural measurements intersect in three-dimensional space. We now highlight a few directions and challenges in the mapping of neural circuits across the larval zebrafish brain.

The generation of complex behaviors, such as decision-making when confronted with competing^33^ or ambiguous^34^ stimuli, requires intricate polysynaptic interactions; their investigations thus require, at the very least, knowledge of both neurochemical identity and connectivity across multiple brain regions. Current connectivity measurements in atlases, which consist mostly of single-cell tracings that begin and terminate in different regions, provide a good starting point to piece together distant cell populations that, for instance, could be identified based on functional criteria, such as their correlated activity with ongoing stimuli or behavior. Given sufficiently detailed functional measurements, atlases can provide experimentalists with narrowed-down circuit hypotheses that account for the observed neuronal dynamics and behaviors. As these databases continue to grow, however, the manual interrogation of thousands of markers and reconstructed cells becomes very tedious, and efforts are underway to find optimal search algorithms and modeling frameworks to efficiently provide users with candidate circuit models.^23^ Hypothetical circuits derived from atlases will then necessarily have to be confronted with functional and structural validations from, for instance, post-hoc screenings of molecular labels,^35^ opsin-based optogenetics,^36^^,^^37^ or viral-based retrograde tracing,^38^ as the true connectivity or colocalization between markers can only be inferred experimentally.

In their current state, atlases are mostly built on optical measurements at the cellular resolution. It is possible to reach the synaptic scale, in whole brain reconstructions, by genetically expressing synaptic markers and tiling the brain at an increased imaging resolution.^39^ Reconstructions of the larval zebrafish brain at the nanometric scale using electron microscopy (EM) have also been conducted,^22^^,^^24^ and these datasets have been incorporated into current atlases. The co-registration of LM and EM measurements offers an unparalleled opportunity to generate more elaborate and constrained circuit hypotheses, although the full reconstruction of these volumes is unfinished and will likely require extensive human intervention and time.^40^

Although the larval zebrafish neuroscience field has been dominated by impressive microscopy studies, comparatively few electrophysiological characterizations have been made, which can be attributed to, among other factors, the substantially greater yield and momentum of optical methods.^10^ Yet, intrinsic electrophysiological properties remain arguably the most important measurements required to constrain models and properly understand neuronal and network functions.^41^^,^^42^ The mouse field has excelled in this regard, benefiting notably from recent developments in multi-channel silicon probes capable of extracellular recordings from hundreds of cells,^43^^,^^44^ as well as large-scale endeavors from multiple institutions to compile multimodal electrophysiological recordings.^45^ As a result, mouse atlases have integrated large datasets comprising paired morphological, electrophysiological, and genetic profiles,^45^^,^^46^ two-photon imaging and multi-channel spike recordings in the visual cortex,^47^^,^^48^ and catalogs of ion channel properties.^49^ As electrophysiological methods become more adapted to the tiny larval zebrafish brain, researchers should look to integrate these indispensable measurements into zebrafish brain atlases, albeit at smaller scales than what is currently achieved in rodents.

The incorporation of other standardized functional measurements into atlases, such as calcium imaging responses to many well-characterized visual stimuli, will require tighter experimental and data sharing norms to achieve robustness and reproducibility.^47^^,^^48^^,^^50^^,^^51^ Current atlases include immediate-early gene expression under various experimental conditions, informing on the spatial distribution of activity but lacking the temporal precision required to tease apart the computations unfolding rapidly during behavior. Neuronal responses of zebrafish larvae to visual stimuli have been mapped using atlases in many calcium imaging studies involving a broad range of microscopy techniques, protocols, and stimulation devices.^52^^–^^54^ Rigorous guidelines for visual stimulation experiments, encompassing both hardware and software considerations, are emerging.^55^^–^^57^ Should calcium imaging measurements integrate publicly available zebrafish atlases, stimulus tuning and functional properties of cells could notably provide strong functional constraints or validations in large-scale circuit models.^58^^–^^60^

The 6 DPF stage has thus far been the dominant time point used to study innate and mostly sensory-driven behaviors, yet more complex behaviors like social interactions^61^ and associative learning^62^ emerge and become more robust in the following weeks. As imaging tools become tailored to probe functionally the larger juvenile zebrafish brain, atlases will have to be replicated at these increasingly studied developmental periods, during which the zebrafish brain remains advantageously accessible for studying the neuronal basis of such behaviors.^63^^,^^64^ A few atlases have already been established in adult zebrafish, including the recent adult zebrafish brain atlas (AZBA)^26^ or a 3D MRI atlas,^25^ though the optical accessibility and whole-brain sampling, along with reduced experimental throughput, are hampered at this stage.

Thus far, our review has focused on mapping the brain, yet expanding atlas boundaries to encompass the entire body of the animal remains an intriguing possibility. The highly accessible larvae offer an unparalleled opportunity to reconstruct^22^ and study spinal locomotor networks,^65^^,^^66^ peripheral sensory systems,^67^ and a wide array of brain-body interactions,^68^ the latter being an increasingly acknowledged topic across the field of neuroscience. Although standard procedures still need to be established to properly image the entire animal, generating a whole-body atlas at cellular resolution is likely to become vital as the field grows to study the intricate relationships between brain, heart,^69^ and gut,^70^ among multiple organs that can be readily imaged in transparent developing larvae.

It should be emphasized that brain atlases are statistical representations of the brain derived from hundreds or thousands of representative specimens, which display high behavioral variability that stems from their equally variable nervous systems.^71^ Owing to differences in brain growth rates and neuronal migrations, the precise location of genetically defined cells most likely varies across fish, and atlases only offer probabilistic maps of the most densely labeled locations in such cases. At the other end of the spectrum, EM reconstructions are derived from one or a few specimens only. The statistical limitations of intersecting multimodal datasets, with different imaging protocols, must be well acknowledged by atlas contributors before reaping the benefits of a very powerful data sharing platform and hypothesis generation tool for neuroscience.

## 5 Conclusion

The small and optically transparent larval zebrafish brain has been the landscape of impressive collective efforts to map the neuronal networks that convey sensory inputs to behavioral outputs. Several brain atlases have been developed to provide an anatomical and numerical scaffold for whole-brain calcium imaging studies, and these resources are continuously expanding due to the accessibility of exhaustive measurements in the larval brain. Numerous challenges emerge when cramming information to the greatest extent into a single three-dimensional volume, and though a complete reconstruction may never be obtained for each developmental stage, the contributions of a growing community of researchers, along with technological progress and increasingly standardized imaging protocols, will undoubtedly push these resources closer to this ideal goal.
